# Supplementary material for: Long-Term Elite Controllers of HIV-1 Infection Exhibit a Deep Perturbation of Monocyte Homeostasis
Source: Int J Mol Sci. 2025 Apr 22;26(9):3926. doi: 10.3390/ijms26093926 (PMC12071947; doi:10.3390/ijms26093926)
Supplement: Supplementary file 1 [file ijms-26-03926-s001.zip › ijms-3484775-supplementary.pdf]

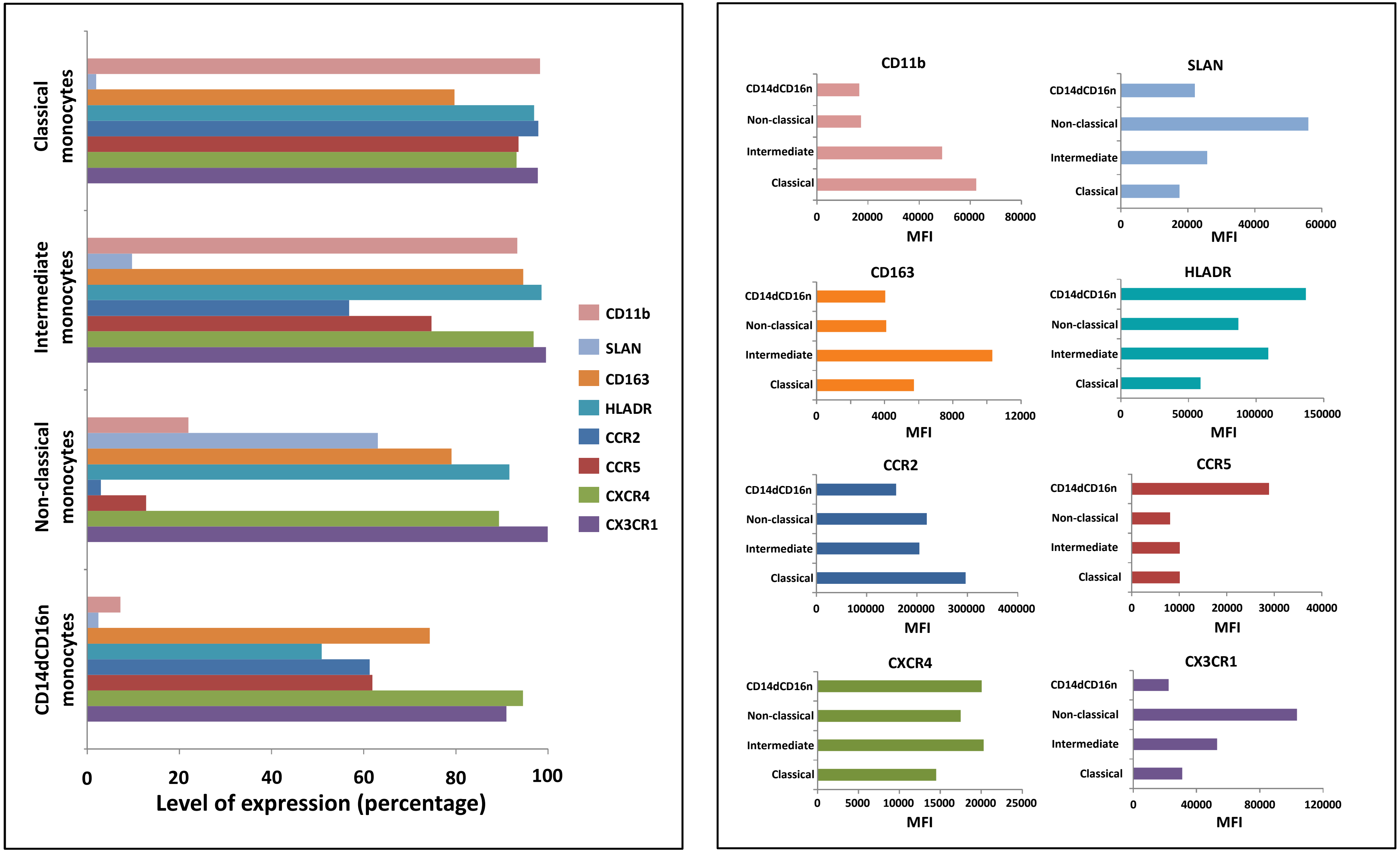

Supplementary figure S1. Bar graphs showing: **Left panel)** the level of expression (expressed as percentage of positive cells) of each single marker by the four different subsets of monocytes in the UC reference study group; **Right panel)** the intensity of expression (expressed as mean fluorescence intensity units; MFI) of each single marker by the four different subsets of monocytes in the UC reference group.

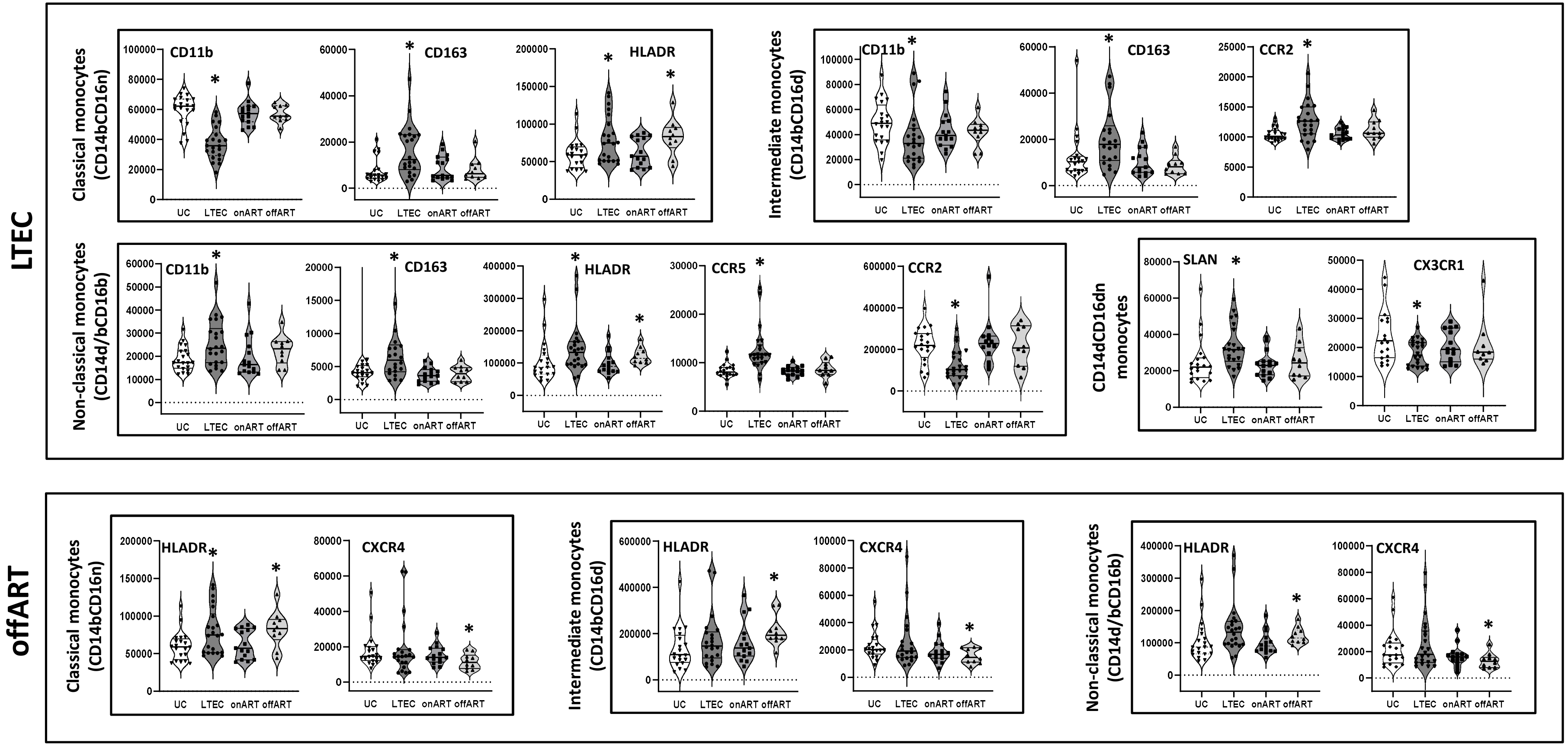

Supplementary Figure S2. Violin-plots graphs showing the expression levels of each single marker by different monocyte subset in the different study groups. Upper plots show those single markers showing significantly different expression in the LTEC group (as compared to UC group), and lower plots show those single markers showing significantly different expression in the offART group (as compared to UC group). Y-axis in the plots represents the intensity of expression (MFI: mean fluorescence intensity units). (*): $p<0.05$ with respect to UC group.

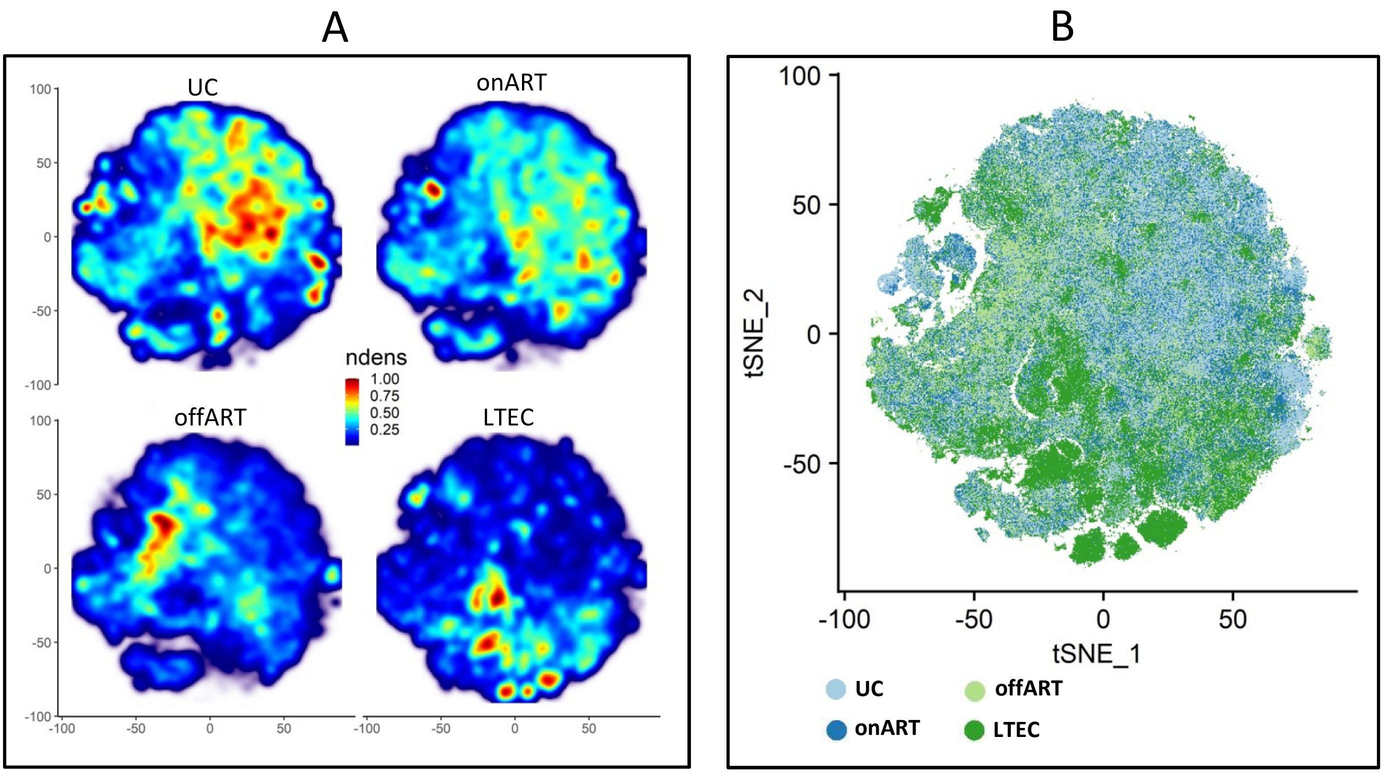

Supplementary figure S3. A) tSNE maps colored by normalized density of events and split by study group. Maps colored by density help us to visualize where events (monocytes) are more condensed in each study group and thus revealing inter-group differences in the distribution of events across the tSNE map. Normalized densities (ndens) were scaled to a maximum of 1 in each map, so that we can compare between different study groups independently of the number of samples analyzed (and thus the number of events) in each group. **B)** tSNE map showing the distribution of events (monocytes) across the map according to the study group (events from the different study groups are color-coded as shwon in the figure). An uneven distribution of the study groups across the map can be observed with events of certain study groups more abundant in certain areas of the map.

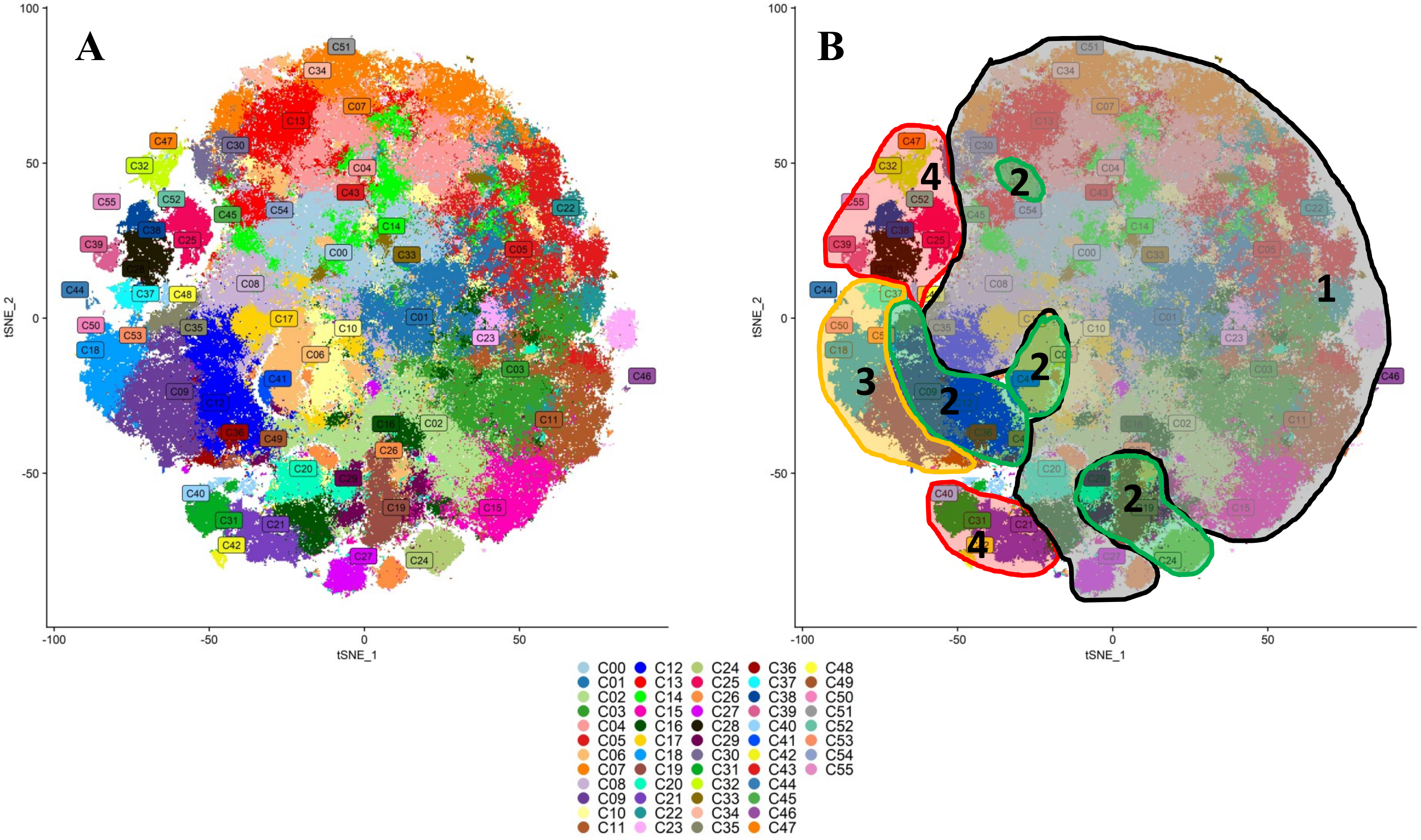

Supplementary figure S4. A) Louvain clustering of tSNE map, showing 56 different clusters of monocytes. The panel at the bottom indicates the color-coding of each cluster. **B)** groups of monocyte clusters (numbered 1-4) according to the expression of CD14 and CD16 markers: group 1: clusters formed by CD14bCD16n monocytes (classical monocytes); group 2: clusters formed by a mixture of CD14bCD16d monocytes (intermediate monocytes) and classical monocytes; group 3: clusters formed by CD14d/bCD16b monocytes (non-classical monocytes); group 4: clusters formed by CD14dCD16n monocytes.

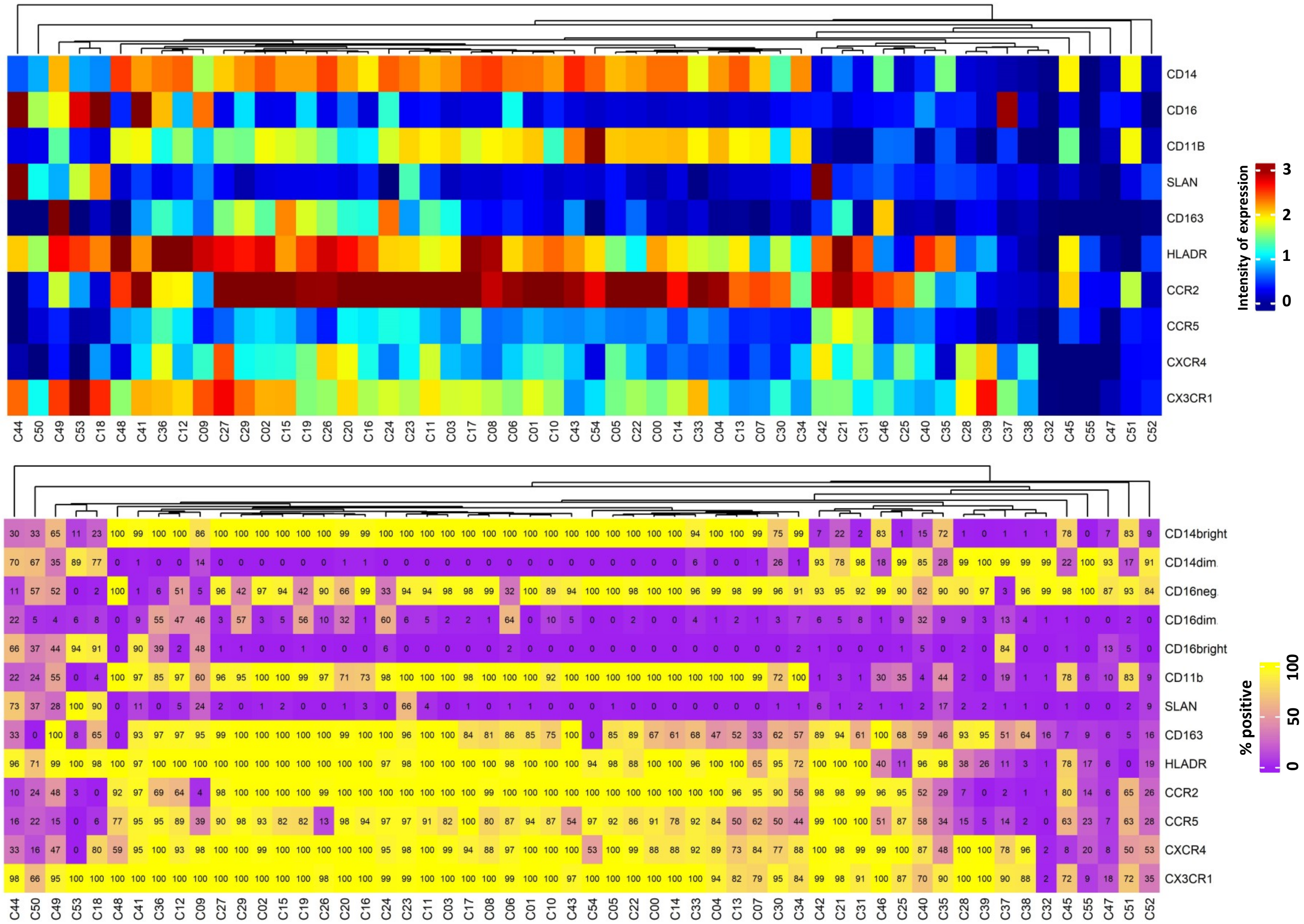

Supplementary figure S5. Top) Heatmap representing the level of expression of markers by each specific cluster of monocytes (cluster fingerprints). Relative level of expression for each marker is color-coded from deep blue (lowest level of expression) to deep red (highest level of expression). The dendrogram at the top of the map shows the grouping of clusters by similarity of markers expression. **Bottom)** Heatmap representing the percentage of positive events for a specific marker in each cluster. Positivity for each marker was defined based on a manual gating (as explained in material and mehods). Level of positivity is color-coded from deep purple (lowest level of positivity) to yellow (highest level of positivity). Number inside each cell of the map indicates the percentage of positive events for a specific marker in a specific cluster. The dendrogram at the top of the map shows the grouping of clusters by similarity of percentage of positivity for each marker. In both maps, each column represents a specific cluster and each file a specific marker.

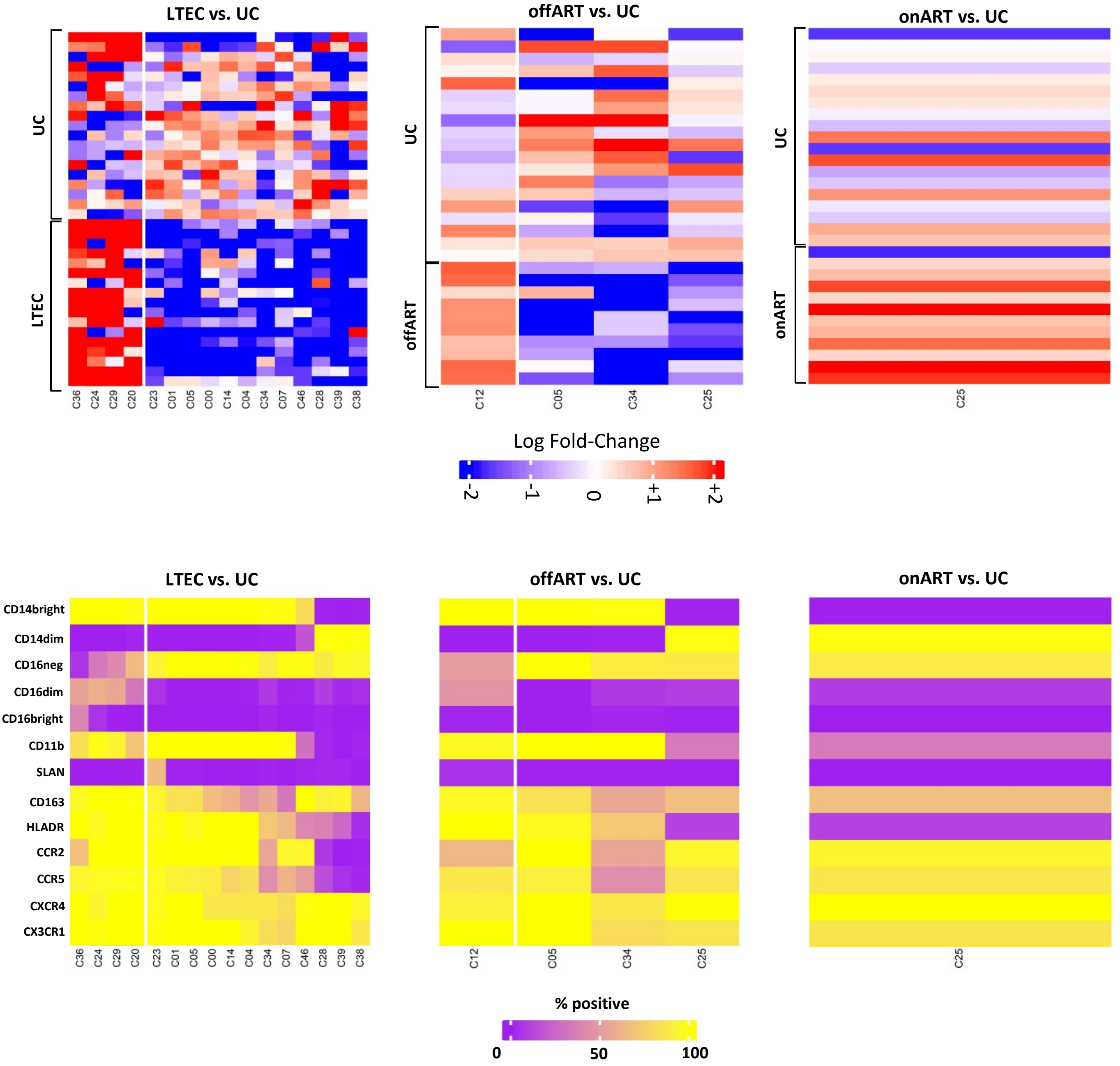

Supplementary figure S6. Top) Heatmaps of expression level of the clusters of monocytes that are differentially expressed between PLWH groups and UC group. The expression level is expressed as Log_2Fold-change using the median value in the UC group as reference. Blue color indicates a diminished expression and red color an increased expression in each individual sample compared to the median value obtained in UC samples. In the heatmaps, each column represents a cluster and each row represents a sample. **Bottom)** Heatmaps representing the level of positivity (expressed as percentage of positive events) for a specific marker in each of the clusters differentially expressed in PLWH groups compared to UC group. Positivity for each marker was defined based on a manual gating (as explained in material and mehods). Level of positivity is color-coded from deep purple (lowest level of positivity) to yellow (highest level of positivity). In the heatmaps, each column represents a cluster and each row represents a marker.

Supplementary table S1. Levels of monocyte clusters showing significant differences (adjusted p-value<0.05) between study groups.

		Study group				
**Monocytes subset**	**Cluster**	**UC**	**LTEC**	**offART**	**onART**	**p<0.05**
**Classical monocytes (CD14bCD16n)**	C00	**7.87** [5.8-11.6]	**1.39** [0.34-3.98]	**9.15** [7.3-15.7]	**6.02** [3.5-8.3]	a, d, e
	C01	**8.7** [4.2-13.7]	**1.0** [0.29-1.87]	**3.96** [1.4-5.8]	**5.3** [2.7-11.8]	a, d, e
	C04	**7.84** [3.5-9.1]	**1.37** [0.5-3.4]	**7.21** [4.6-8.5]	**5.0** [3.7-9.3]	a, d, e
	C05	**7.36** [4.5-10.4]	**0.76** [0.31-1.09]	**2.44** [0.39-3.20]	**5.51** [3.72-10.5]	a, b, e
	C07	**5.13** [3.5-6.6]	**1.86** [0.8-3.61]	**1.85** [1.16-3.43]	**3.49** [2.39-7.0]	a
	C08	**2.91** [2.0-3.7]	**1.72** [0.85-3.02]	**6.39** [4.4-13.8]	**2.7** [1.7-4.2]	d
	C13	**2.91** [1.7-4.8]	**1.58** [0.9-2.5]	**4.27** [2.5-7.4]	**2.51** [1.8-3.9]	d
	C14	**4.27** [1.9-5.5]	**1.39** [0.5-1.4]	**3.39** [1.6-4.9]	**4.12** [2.8-4.9]	a, e
	C23	**1.12** [0.8-1.9]	**0.27** [0.2-0.5]	**1.13** [0.4-3.8]	**1.01** [0.6-1.5]	a, d, e
	C34	**1.07** [0.4-2.2]	**0.11** [0.05-0.3]	**0.16** [0.08-0.5]	**0.52** [0.4-0.9]	a, b, e
	C46	**0.03** [0.01-0.06]	**0** [0-0.01]	**0.01** [0.01-0.02]	**0.02** [0.01-0.03]	a
**Intermediate monocytes (CD14bCD16d)**	C12	**2.0** [1.84-3.23]	**2.32** [1.72-3.81]	**5.33** [4.3-6.6]	**2.73** [2.3-4.4]	b, d, f
	C20	**0.1** [0.08-0.4]	**1.27** [0.2-3.1]	**0.09** [0.04-0.2]	**0.19** [0.04-0.3]	a
	C24	**0.01** [0-0.05]	**0.33** [0.03-0.6]	**0.03** [0.02-0.2]	**0.02** [0-0.08]	a
	C29	**0.02** [0.01-0.2]	**0.93** [50.2-1.5]	**0.08** [0.05-0.2]	**0.19** [0.03-0.3]	a, d, e
	C36	**0.02** [0.01-0.05]	**0.22** [0.09-1.0]	**0.04** [0.02-0.06]	**0.02** [0.01-0.05]	a, d, e
**CD14dCD16n monocytes**	C25	**0.9** [0.7-1.4]	**0.51** [0.4-1.1]	**0.35** [0.2-0.5]	**1.69** [1.4-3.4]	b, c, e, f
	C28	**0.69** [0.2-1.3]	**0.16** [0.06-0.3]	**0.26** [0.1-0.7]	**0.5** [0.2-1.4]	a, e
	C38	**0.43** [0.2-1.1]	**0.04** [0.01-0.1]	**0.14** [0.05-0.3]	**0.36** [0.2-0.5]	a, e
	C39	**0.01** [0-0.1]	**0** [0-0]	**0** [0-0]	**0.01** [0-0.2]	a, e

Levels are given as **median** [Q1-Q3] and expressed as percentage of total monocytes
a: LTEC vs. UC; **b:** offART vs. UC; **c:** onART vs. UC; **d:** LTEC vs. offART; **e:** LTEC vs. onART; **f:** offART vs. onART

A

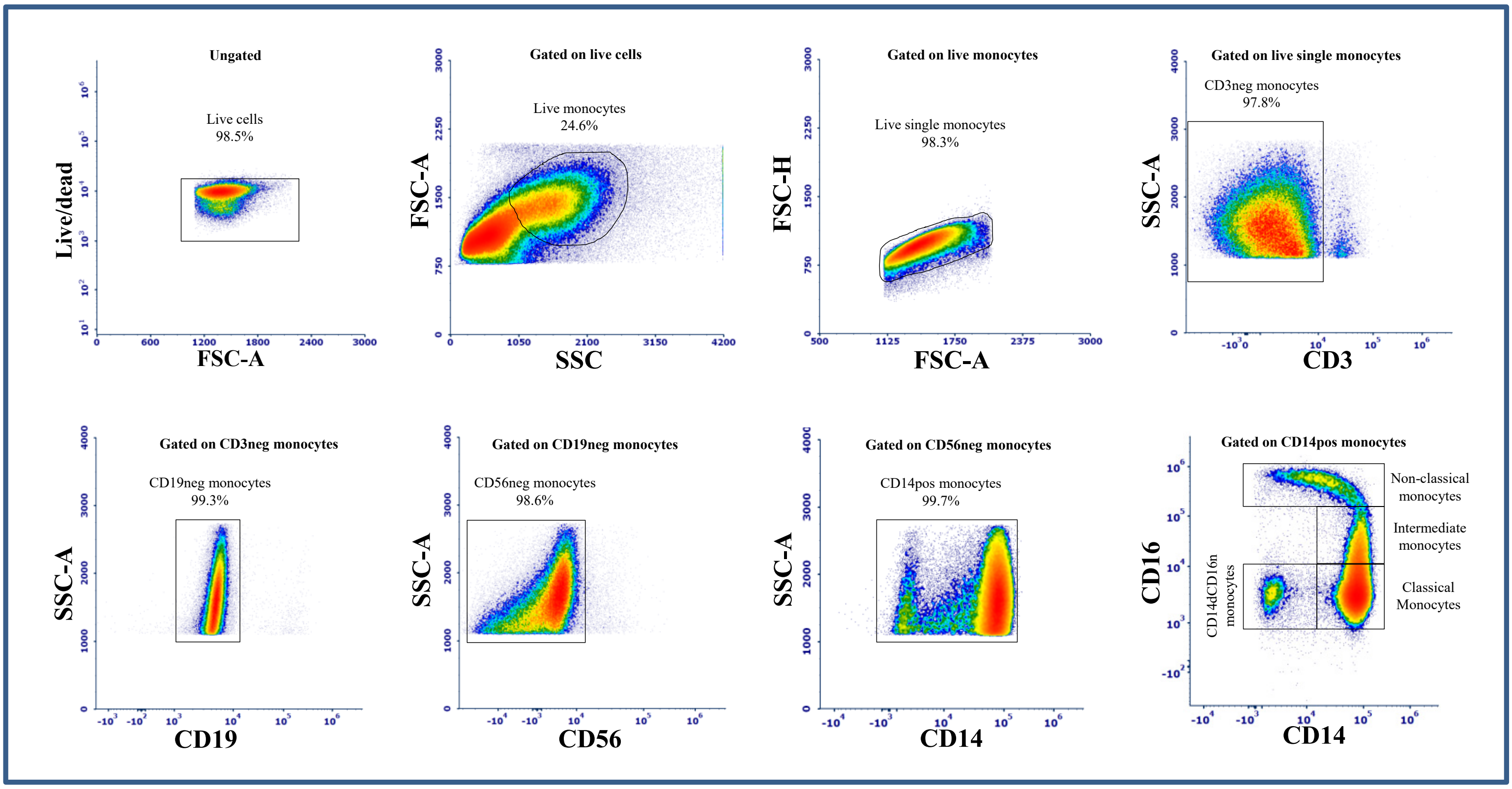

B

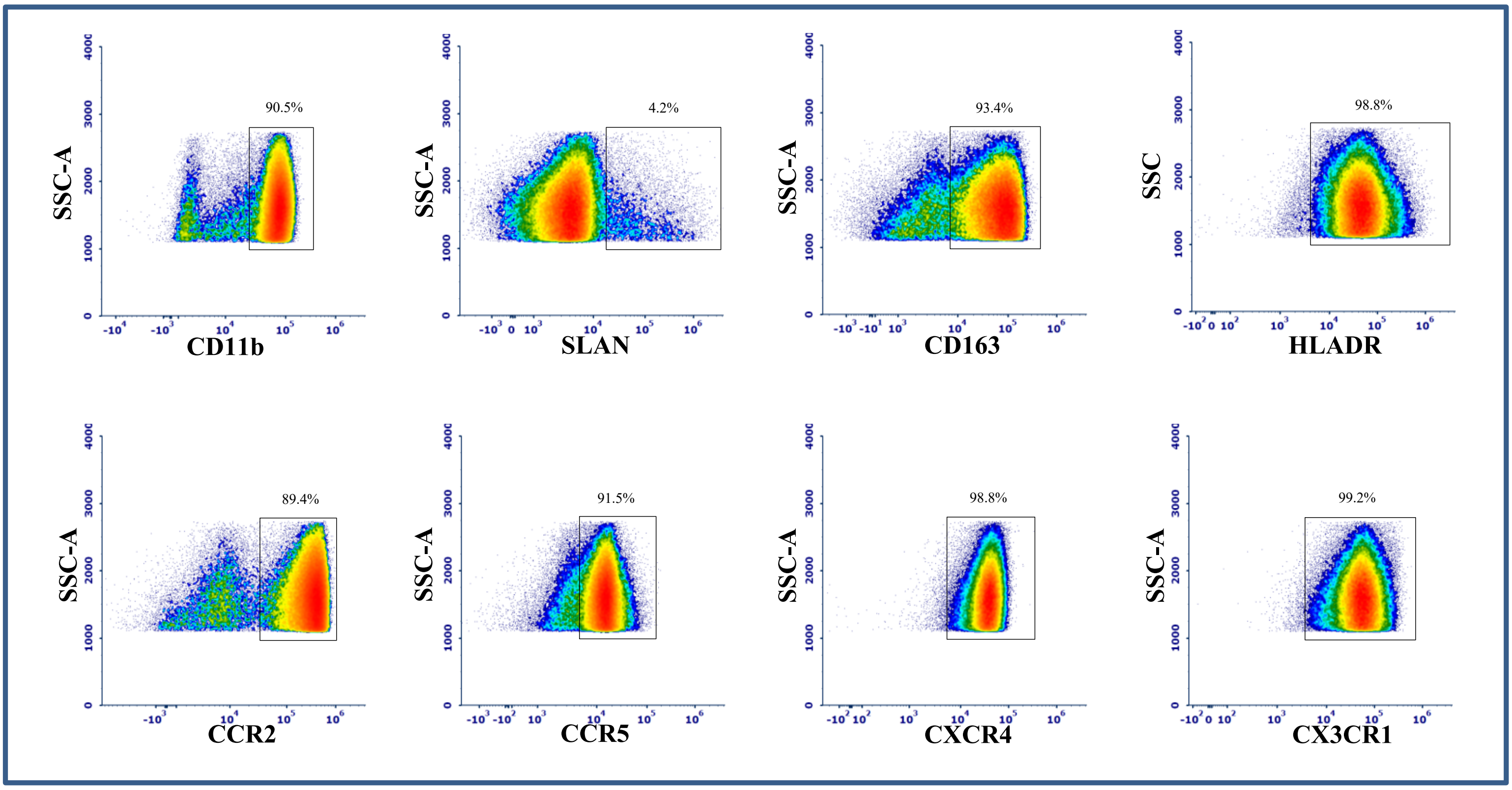

Supplementary figure S7. Flow cytometry example showing: **A)** the sequential strategy of gating for monocytes (dot-plots graphs in upper two rows). The sequential gating included the next steps: gating of live cells using the LiveDead marker, gating of live monocytes based on FSC and SC parameters, gating of live single monocytes based on FSC-A and FSC-H parameters, gating of CD3- monocytes using CD3 marker, sequential gating of CD19-, CD56- monocytes and finally gating of CD14+ monocytes and of different subsets of monocytes based on relative expression (bright(b), dim(d) or negative(n)) of CD14 and CD16: CD14bCD16n (classical monocytes), CD14bCD16d (intermediate monocytes), CD14d/bCD16b (non-classical monoytes) and CD14dCD16n monocytes; **B)** the expression of different markers on total monocytes with the gates for positivity of each marker (lower two rows).

Clinical Centers and research groups which contribute to ECRIS cohort

Clinical centers:

Hospital Universitario de Valme (Sevilla): Juan Antonio Pineda, Eva Recio Sánchez, Fernando Lozano de León, Juan Macías, José Carlos Palomares, Manuel Parra, Jesús Gómez-Mateos.

Hospital General Universitario Santa Lucía (Cartagena): Onofre Juan Martínez-Madrid, Francisco Vera, Lorena Martínez.

Hospital Clinic de Barcelona (Barcelona): José M. Miró, José Luis Blanco, Felipe García, Esteban Martínez, Josep Mallolas, Montserrat Laguno, María Martínez, Berta Torres, Lorna Leal, Ainoa Ugarte, Flor Etcheverry, Irene Fernández.

Hospital General Universitario de Alicante (Alicante): Joaquín Portilla, Esperanza Merino, Sergio Reus, Vicente Boix, Livia Giner, Carmen Gadea, Irene Portilla, Maria Pampliega, Marcos Díez, Juan Carlos Rodríguez, Jose Sánchez-Payá.

Hospital Universitari de Bellvitge (Hospitalet de Llobregat): Daniel Podzamczer, Elena Ferrerm Arkaitz Imaz, Evan Van Den Eyncle, Silvana Di Yacovo, Maria Sumoy.

Hospital Universitario de Canarias (Santa Cruz de Tenerife): Juan Luis Gómez, Patricia Rodríguez, María Remedios Alemán, María del Mar Alonso, María Inmaculada Hernández, Felicitas Díaz-Flores, Dácil García, Ricardo Pelazas.

Hospital Carlos III (Madrid): Vicente Soriano, Pablo Labarga, Pablo Barreiro, Pablo Rivas, Francisco Blanco, Luz Martín Carbonero, Eugenia Vispo, Carmen Solera.

Hospital Universitario Central de Asturias (Oviedo): Victor Asensi, Eulalia Valle, José Antonio Cartón.

Hospital Doce de Octubre (Madrid): Rafael Rubio, Federico Pulido, Mariano Matarranz, Maria Lagarde, Guillermo Maestro, Rafael Rubio-Martín.

Hospital Universitario Donostia (San Sebastián): José Antonio Iribarren, Julio Arrizabalaga, María José Aramburu, Xabier Camino, Francisco Rodríguez-Arrondo, Miguel Ángel von Wichmann, Lidia Pascual Tomé, Miguel Ángel Goenaga, Mª Jesús Bustinduy, Harkaitz Azkune Galparsoro. Maialen Ibarguren, Mirian Aguado.

Hospital General Universitario de Elche (Elche): Félix Gutiérrez, Mar Masiá, Cristina López, Sergio Padilla, Andrés Navarro, Fernando Montolio, Catalina Robledano, Joan Gregori Colomé, Araceli Adsuar, Rafael Pascual, Federico Carlos, Maravillas Martinez.

Hospital Germans Trías i Pujol (Badalona): Roberto Muga, Jordi Tor, Arantza Sanvisens.

Hospital General Universitario Gregorio Marañón (Madrid): Juan Berenguer, Juan Carlos López Bernaldo de Quirós, Pilar Miralles, Isabel Gutiérrez, Margarita Ramírez, Belén Padilla, Paloma Gijón, Ana Carrero, Teresa Aldamiz-Echevarría, Francisco Tejerina, Francisco Jose Parras, Pascual Balsalobre, Cristina Diez.

Hospital Universitari de Tarragona Joan XXIII, IISPV, Universitat Rovira i Virgili (Tarragona): Francesc Vidal, Joaquín Peraire, Consuelo Viladés, Sergio Veloso, Montserrat Vargas, Miguel López-Dupla, Montserrat Olona, Alba Aguilar, Joan Josep Sirvent, Verónica Alba, Olga Calavia.

Hospital Universitario La Fe (Valencia): Marta Montero, José Lacruz, Marino Blanes, Eva Calabuig, Sandra Cuellar, José López, Miguel Salavert.

Hospital Universitario La Paz/IdiPaz (Madrid): Juan González, Ignacio Bernardino de la Serna, José Ramón Arribas, María Luisa Montes, Jose Mª Peña, Blanca Arribas, Juan Miguel Castro, Fco Javier Zamora, Ignacio Pérez, Miriam Estébanez, Silvia García, Marta Díaz, Natalia Stella Alcáriz, Jesús Mingorance, Dolores Montero, Alicia González, Maria Isabel de José.

Hospital de la Princesa (Madrid): Ignacio de los Santos, Jesús Sanz, Ana Salas, Cristina Sarriá, Ana Gómez.

Hospital San Pedro-CIBIR (Logroño): José Antonio Oteo, José Ramón Blanco, Valvanera Ibarra, Luis Metola, Mercedes Sanz, Laura Pérez-Martínez.

Complejo Hospitalario de Navarra (Pamplona): María Rivero, Marina Itziar Casado, Jorge Alberto Díaz, Javier Uriz, Jesús Repáraz, Carmen Irigoyen, María Jesús Arraiza.

Hospital Parc Taulí (Sabadell): Ferrán Segura, María José Amengual, Gemma Navarro, Montserrat Sala, Manuel Cervantes, Valentín Pineda, Victor Segura, Marta Navarro, Esperanza Antón, Mª Merce Nogueras.

Hospital Ramón y Cajal (Madrid): Santiago Moreno, José Luis Casado, Fernando Dronda, Ana Moreno, María Jesús Pérez Elías, Dolores López, Carolina Gutiérrez, Beatriz Hernández, Nadia Madrid, Angel Lamas, Paloma Martí, Alberto de Diaz, Sergio Serrano, Lucas Donat.

Hospital Reina Sofía (Murcia): Alfredo Cano, Enrique Bernal, Ángeles Muñoz.

Hospital San Cecilio (Granada): Federico García, José Hernández, Alejandro Peña, Leopoldo Muñoz, Jorge Parra, Marta Alvarez, Natalia Chueca, Vicente Guillot, David Vinuesa, Jose Angel Fernández.

Centro Sanitario Sandoval (Madrid): Jorge Del Romero, Carmen Rodríguez, Teresa Puerta, Juan Carlos Carrió, Cristina González, Mar Vera, Juan Ballesteros.

Hospital Son Espases (Palma de Mallorca): Melchor Riera, María Peñaranda, María Leyes, Mº Angels Ribas, Antoni A Campins, Carmen Vidal, Leire Gil, Francisco Fanjul, Carmen Matinescu.

Hospital Universitario Virgen del Rocío (Sevilla): Manuel Leal, Pompeyo Viciana, Luis Fernando López-Cortés, Nuria Espinosa.

Research groups:

IIS-Fundación Jimenez Díaz, UAM: José Miguel Benito, Norma Rallón, Clara Restrepo, Alfonso Cabello, Miguel Gorgolas.

Infección viral e Inmunidad. ISCIII: Salvador Resino, Veronica Briz, Maria Angeles Jimenez, Maria Sonia Vazquez, Amanda Fernandez, Pilar García.

Hospital General Universitario Gregorio Marañón: Maria Angeles Muñoz, Javier Sanchez Rodriguez, Jose Luis Jimenez, Daniel Sepúlveda, Isabel García Merino, Irene Consuegra.

Hospital Clinic-IDIBAPS: Agathe León, Sonsoles Sánchez, Mireia Arnedo, Montserrat Plana, Nuria Climent, Felipe García.

Hospital Joan XXIII: Paco Vidal, Esther Rodriguez-Gallego, Consuelo Viladés, Joaquin Peraire

Centro Sandoval: Jorge Del Romero, Carmen Rodríguez, Mar Vera.

Fundación IrsiCaixa: José Esté, Esther Ballana, Miguel Angel Martinez, S Franco,María Nevot.

Hospital Ramón y Cajal: Alejandro Vallejo, Beatriz Sara Sastre, Santiago Moreno.

Virologia Molecular ISCIII: Maria Pernas, Concepción Casado, Cecilio López Galíndez

Inmunopatología del SIDA, ISCIII: Laura Capa, Mayte Perez-Olmeda, Pepe Alcami

Mutación y evolución de virus. Univ. Valencia: Rafael Sanjuán, José Manuel Cuevas

Hospital Virgen del Rocío: Ezequiel Ruiz-Mateos, Beatriz Dominguez-Molina, Laura Tarancón-Diez, Mohamed Rafii-El-Idrissi Benhnia, Maria José Polaino, Miguel Genebat, Pompeyo Viciana, Manuel Leal.

Hospital 12 de Octubre: Rafael Delgado, Olalla Sierra

Universidad de la Laguna: Agustín Valenzuela-Fernández.

Staining protocol for flow cytometry

One million of PBMCs were washed with 2 mL of phosphate-buffered saline (PBS) and stained for surface markers by sequential incubation, as follows: firstly cells were incubated with Live/dead fixable blue viability dye for 15 minutes in the dark at room temperature (RT). Secondly, cells were washed with 2ml of PBS and sequentially incubated in 100 μL with the next reagents: firstly for 10 minutes at RT in the dark with Trustain Fcx (to block the Fc receptor expressed by monocytes); secondly for another 10 minutes at RT in the dark with a mix containing the optimal amount of the next antibodies: anti-CXCR4, anti-CCR2, anti-CCR5, anti-CX3CR1; plus 20 μL of brilliant satin buffer; thirdly for 30 minutes at RT in the dark with a second mix containing the optimal amount of the rest of antibodies included in the panel. After this incubation, cells were washed with 2 mL of PBS containing 0.5% of fetal bovine serum (FBS) and resuspended in 150 μL of PBS plus 0.5% of FBS for acquisition in an Aurora spectral flow cytometer (Cytek Biosciences, USA).

Unsupervised analysis of flow cytometry data

Flow cytometry data from each study participant were manually pre-gated using FCS Express 7 software as described above. Using the monocytes gate (CD14+ cells), each flow cytometry data file was converted to a new file containing only monocyte events. Next, these new data files were annotated using FCS Express 7 to include the information about the single positivity/negativity of each monocyte event contained in the file for each of the 10 different cell surface markers analyzed in monocytes (markers included in **supplementary table 2,** excluding CD3, CD16, and CD56, that were used for excluding contaminating T cell, B cells and NK cells respectively). Of these 10 different markers, CD14 and CD16 markers were used to define different monocyte subsets based on the relative expression of these markers (using three different categories of expression for CD16 marker and two categories of expression for CD14 marker)

All pre-gated data files containing only monocytes were included for unsupervised multidimensional analysis, including batch correction, dimensionality reduction, and clustering analysis. R software (version 4.1.1) was used for implementation of the automated pipeline. The workflow for this analysis is shown in **supplementary figure 8**.

First, batch effects were corrected using the CytoNorm algorithm. By taking advantage of the existence of the same control sample acquired in each batch or experiment along with the rest of the samples, non-biological variability was estimated and removed from the data before their unbiased analysis. Each file was subsampled to 15.000 cells to facilitate proper processing of all samples at once, and to balance the contribution of each sample to the total number of cells analyzed. After transforming the data with the

arcsinh function (cofactor=6000), marker expression levels were jointly scaled via Zscore normalization. The whole panel of fluorescent markers (except CD3, CD16 and CD56 markers) was used in the downstream analysis using Seurat R package. For the detection of cell clusters sharing a similar expression fingerprint, we used Louvain's algorithm, which identifies clusters by modularity optimization from a shared nearest neighbor graph (SNN). A level of cluster granularity corresponding to a resolution parameter value of 2.0 was used. To visualize single-cell measurements and clustering results, we used 2-dimensions representations obtained with nonlinear dimensionality reduction techniques. T-distributed stochastic neighbor embedding (tSNE) maps were obtained using FFT-accelerated Interpolation-based t-SNE (FIt-SNE) algorithm (Parameters: perplexity=30, theta=0.5, distance=Euclidean, rest of default parameters). Cell frequencies in each cluster were computed and compared between each pair of study groups using wilcoxon test. P-values were corrected using Holm method. We also computed Fold Change with respect to a reference group (typically UC group) to represent the relative changes in clusters proportions between groups.

Supplementary Table S2: Monoclonal antibodies and fluorochromes used in the study

Antibody	Fluorochrome	Clone	Provider
CD3	AF700	OKT3	Biolegend
CD16	AF488	3G8	Biolegend
CD56	BV605	5.1H11	Biolegend
CD19	BV480	SJ25C1	BD Biosciences
CD14	BV711	63D3	Biolegend
CD11b	PerCP-CY5.5	ICRF144	Biolegend
HLADR	APC-FIRE750	L243	Biolegend
CD163	AF647	GHI/61	Biolegend
SLAN(M-DC8)	PE	REA1050	Miltenyi
CCR2(CD192)	PE-DAZZLE	K036C2	Biolegend
CCR5 (CD195)	PE-CY5	2D7/CCR5	BD Biosciences
CXCR4 (CD184)	BV421	12G5	Biolegend
CX3CR1	AF647	2A9-1	Biolegend

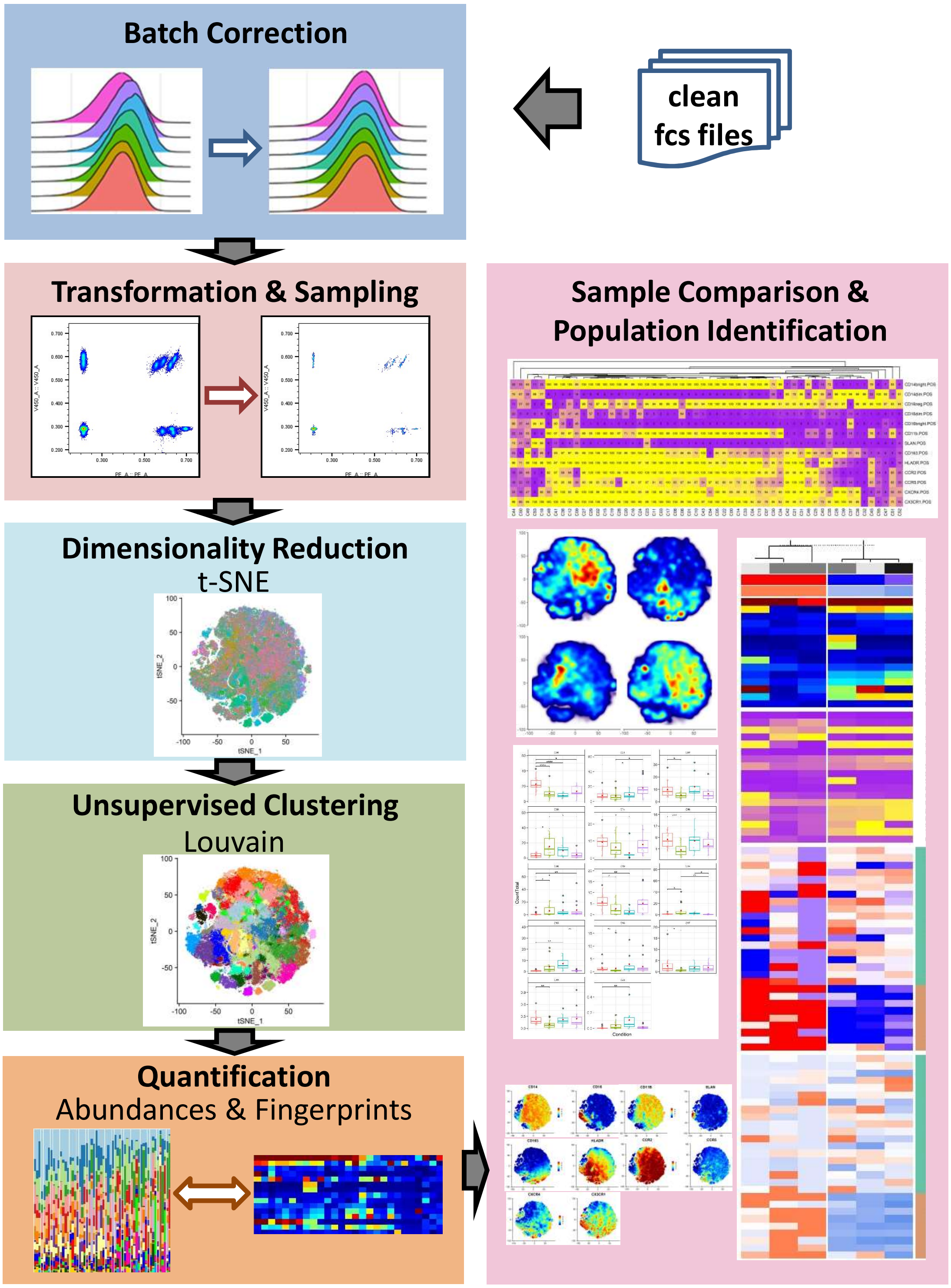

Supplementary figure S8. Pipeline for multidimensional analysis of flow cytometry data.
